# Mediation of executive functions in the relationship between motor skills and psychosocial health in preschool children

**DOI:** 10.1016/j.jesf.2025.04.002

**Published:** 2025-04-03

**Authors:** Sen Li, Yang Song, Qingwen Zhang, Zhen Wang

**Affiliations:** a School of Physical Education and Health, Shanghai Lixin University of Accounting and Finance, Shanghai, China; b Rizhao Sports School, Rizhao, 276800, China; c School of Physical Education, Shanghai University of Sport, Hengren Road 200, Shanghai, 200438, China

## Abstract

**Purpose:**

Early motor skills develop alongside executive functions and psychosocial health. However, the interaction between these elements in early childhood is not well-studied. This study aimed to examine whether executive functions mediate the relationship between motor skills and psychosocial health.

**Methods:**

A total of 452 children (mean age = 6.14 ± 0.29 years, 48.9 % female) were included in this cross-sectional study. The Movement Assessment Battery for Children-Second Edition (MABC-2) was used to assess motor skills. Executive functions were measured using the Go/No-Go Test, Dimensional Change Card Sort Test, and List Sorting Working Memory Test from the Early Years Toolbox (ages 3–7). Social skills and problem behaviors were assessed using the preschool version of the Social Skills Improvement System Rating Scale (SSIS-RS). Structural equation modeling (SEM) with maximum likelihood estimation was employed to examine the mediating role of executive functions.

**Results:**

Gross motor skills were positively associated with inhibition (β = 0.41, p < 0.01), shifting (β = 0.20, p < 0.01), working memory (β = 0.30, p < 0.01), social skills (β = 0.50, p < 0.05), and negatively associated with problem behaviors (β = −0.23, p < 0.05). Inhibition (β = 0.107, p < 0.001) and shifting (β = −0.018, p < 0.05) mediated the relationship between gross motor skills and social skills. Additionally, inhibition (β = −0.086, p < 0.001) and shifting (β = 0.019, p < 0.05) mediated the relationship between gross motor skills and problem behaviors. Fine motor skills were positively associated with inhibition (β = 0.35, p < 0.01), shifting (β = 0.16, p < 0.01), and working memory (β = 0.21, p < 0.01), but not significantly related to social skills (β = 0.08, p > 0.05) or problem behaviors (β = 0, p > 0.05). Inhibition (β = 0.144, p = 0.001) mediated the relationship between fine motor skills and social skills, while both inhibition (β = −0.102, p = 0.001) and shifting (β = 0.014, p = 0.041) mediated the relationship between fine motor skills and problem behaviors.

**Conclusion:**

Executive functions significantly mediate the association between motor skills and psychosocial health in preschool children. Future experimental studies are required to examine causality in young children.

## 1 Introduction

Motor skills are a critical aspect of development in early childhood and can be classified into gross and fine motor skills. It has been known that motor skills have benefits for physiological and psychosocial development in preschool children.^1^ Gross motor skills refer to the ability to utilize large muscle groups to perform activities like running, climbing, and catching, comprising locomotor skills, object control skills, and balance.^2^ These capabilities offer various benefits to youth, including physiological (e.g., healthy weight status), psychological (e.g., self-perception), and behavioral (e.g., increased physical activity).^3^ In contrast, fine motor skills pertain to smaller muscle groups, enabling precise control movements of hands and feet.^2^ They are crucial for tasks requiring visual-motor integration.^4^ Both gross and fine motor skill development is interconnected, affecting cognitive functions (e.g., executive functions) and psychosocial health (e.g., social interactions).^5^, ^6^, ^7^

Executive functions can be defined as higher-order cognitive processes that are employed when automatic or instinctive responses are insufficient or inappropriate.^8^ These include working memory, which entails retaining and manipulating information; cognitive flexibility, or the ability to stay focused while adapting to changing stimuli; and inhibitory control, the capacity to override automatic responses in performing more suitable actions.^8^ The ages between 3 and 6 represent a crucial period for motor, cognitive, and social development. Motor development is closely intertwined with executive function, which involves the regulation of both cognition and emotion, beginning from infancy.^9^ Addressing motor and emotional challenges requires the seamless integration of motor, emotional, and attentional systems, which depends on the simultaneous maturation of their associated neural networks.^10^^,^^11^ As a result, variations in motor development are likely to impact executive functioning and psychosocial health, both of which require effective emotion regulation.^12^

Executive function develops during early childhood and is shaped by environmental and developmental factors. The early years are a crucial period for its growth, although these functions remain somewhat malleable throughout life.^13^ Given the significance of executive function for both early and later academic success, identifying skills that promote its development during preschool is essential.^12^^,^^14^ Research has well documented that motor skills are associated with executive functions in young people.^5^^,^^15^^,^^16^ A recent meta-analysis found the strongest correlations between balance, manual dexterity, and executive functions in preschool children aged 3–12 years.^15^ For example, previous studies have further demonstrated positive associations between motor skills and executive function in preschool children.^17^^,^^18^ This close relationship may be attributed to the existence of shared neural substrates.^19^^,^^20^

The period of early childhood represents a crucial phase in the development of psychosocial health. This can be conceptualized in two distinct ways: the absence of negative characteristics (such as aggression and anxiety) and the presence of positive traits (such as self-esteem and strong friendships).^21^^,^^22^ Psychosocial health includes social skills (e.g., social interaction, self-regulation) and problem behaviors, such as internalizing and externalizing behaviors.^22^ Enhanced social behavior is positively linked to better outcomes in educational contexts, including better classroom functioning, more positive school adjustment, increased levels of academic motivation, and greater engagement in learning.^23^ The transition from preschool to formal schooling and early academic achievement is contingent on the development of key competencies such as cooperation and emotional regulation.^24^ In contrast, externalizing behaviors are associated with difficulties in both academic and social development.^25^^,^^26^ The available evidence suggests that motor skills have a positive impact on psychosocial health in early childhood.^6^^,^^16^ A review of the literature indicates that there is a strong association between motor skill proficiency and social competence in children.^16^ Studies have demonstrated that children who lack proficiency in motor skills often exhibit difficulties in social interactions and may resort to avoiding physical activities as a coping strategy.^27^ Furthermore, existing literature suggests motor competence was positively associated with social competence in younger children.^12^^,^^28^ Specifically, motor skills, particularly object manipulation, support active play and schoolyard activities, which are crucial for peer socialization.^12^ However, most previous research has focused on children with atypical development rather than those with typical development. Evidence from typically developing children may offer valuable insights for educational practices in schools.

The *Learning to Learn* framework emphasizes that physical activity offers opportunities to develop motor skills, enabling children to adapt their movements as their bodies grow.^29^ Early forms of learning, such as motor skill flexibility, may form the foundation for advanced cognitive functions and social behavior.^29^ Social behaviors like self-control, cooperation, and the reduction of hyperactivity or externalizing behaviors require emotional regulation, which overlaps neurobiologically with executive functions like planning and impulse control.^10^^,^^11^ This suggests motor skill development could strengthen executive functions, thereby indirectly supporting psychosocial health in young children. Hill et al.^16^ systematically reviewed evidence linking motor competence to improved executive functions and psychosocial health in preschoolers. However, their work did not explore how executive functions might mediate this relationship—a critical gap. Building on this, Bao et al.^5^ conducted a meta-analysis and identified a modest but consistent correlation (*r* = 0.18, 95 % CI [0.13–0.22]) between motor competence and executive functions in youth. While existing studies broadly agree that motor skills benefit both cognitive and psychosocial outcomes, few have tested whether executive functions explain this connection. For example, preliminary evidence suggests executive functions mediate links between gross motor skills and emotional health in preschoolers,^30^, ^31^, ^32^ but broader psychosocial outcomes (e.g., social skills, internalizing/externalizing behaviors) remain unexplored.

To address this, our study applies the Learning to Learn framework to rigorously investigate executive functions as a potential mediator between motor skills and psychosocial health. This novel approach aims to clarify the mechanisms driving these relationships, offering actionable insights for interventions targeting holistic child development. By leveraging this framework, the research provides key insights into how motor skills impact broader developmental outcomes through cognitive pathways, contributing to both theoretical knowledge and practical advancements in early childhood development. Therefore, the aims of this study were to 1) examine the relationship between motor skills, executive functions, and psychosocial health in younger children; and 2) investigate whether there is a mediating effect of executive functions on the association between motor skills and psychosocial health in this group.

## 2 Materials and methods

The research employed a cross-sectional study, with data collection taking place between March and June 2023. The participants in the current study included typically developing children selected from public kindergartens across three districts in Shanghai, China, using a convenience sampling method. Eligibility criteria for participation were age between 4 and 6 years, regular attendance in kindergarten activities, provision of written consent from a parent or guardian, and verbal assent from the child. Children with physical or intellectual impairments were excluded from the present study, including significant motor developmental delays, color vision deficiencies, chronic illnesses, neurological disorders, or severe cognitive impairments. Before the commencement of the study, approval was sought and granted by the Ethics Committee of Shanghai University of Sports.

## 3 Data collection procedures

All measurements were conducted in a quiet room within the participating kindergartens, ensuring a consistent and distraction-free environment. Research staff, who had undergone the relevant training, administered the assessments during regular school hours. Each child participated in a testing session lasting up to 45 min, with breaks provided as needed. Prior to testing, standardised instructions were given in Mandarin, and children received practice trials to familiarise themselves with the tasks. First, the participants completed the motor skills assessment. Next, they completed the executive functions assessment. Teachers independently completed the SSIS-RS rating scale based on their observations of each child over the preceding month.

## 4 Measures

A total of 452 participants were recruited for the study. The age, sex, and weight status of the subjects were ascertained via the administration of a self-report questionnaire completed by the parents. The participant’s weight and height were assessed on two occasions following established protocols. To calculate the body mass index (BMI), the mean of both measurements of weight and height was used. BMI was calculated by dividing the mean weight (in kilograms) by the square of the mean height (in meters).

***Motor skills*** The Movement Assessment Battery for Children version 2 (MABC-2) was utilized to evaluate motor skills. Previous reliability and validity studies have reported that the MABC-2 was a reliable (ICC >0.9) and valid (I-CVI >0.78) instrument to assess motor skills in Chinese preschool children.^33^ The MABC-2 comprises three subtests: manual dexterity (three tasks), aiming and catching (two tasks), and static and dynamic balance (three tasks). Manual dexterity includes (a) posting coins, (b) threading beads, and (c) drawing trails. Aiming and catching involves catching and throwing a beanbag onto a mat. The balance subtest assesses one-leg balance, heel walking, and mat jumping. These three motor categories are further classified into two groups: fine motor skills (posting coins, threading beads, and drawing trails) and gross motor skills (catching and throwing beanbags, static and dynamic balance). Participants' raw scores on the eight MABC-2 tasks were converted into standard scores, with higher scores indicating superior performance.

***Executive Functions*** The Go/No-Go Test, the Dimensional Change Card Sort Test, and the List Sorting Working Memory Test from the Early Years Toolbox (3–7 years) were used to assess executive functions.^34^ The EYT tools show good internal consistency (Cronbach's alpha 0.80–0.90), strong convergent validity (*r* = 0.50–0.70), and clear developmental sensitivity, making them suitable for assessing abilities in young children.^35^

**Inhibition** was assessed using the EYT Go/No-Go task. Participants tapped the screen to catch a fish (“Go” trials), and refrained from tapping to avoid a shark (“No-Go” trials). The task consisted of 75 stimuli divided into three blocks with 80 % being “Go” trials and 20 % “No-Go” trials, presented in a pseudo-random order to prevent patterns. Before the test, participants completed a training session comprising separate “Go” and “No-Go” practice trials, followed by a mixed block of practice with auditory feedback. This ensured participants understood the task rules. During the test, stimuli were presented for 1500 ms with a 1000-ms interstimulus interval. Consecutive “No-Go” trials were limited to a maximum of two to maintain a robust response tendency. The inhibitory control score was calculated as the product of accuracy rates for “Go” and “No-Go” trials, with a maximum score of 1, reflecting the participant's ability to balance response tendency and inhibition.

***Shifting*** We used the EYT Card Sorting task to assess shifting. The participants were instructed to sort a set of cards, which included images of objects such as a red bunny or a blue ship, into one of two bins, labeled with either a blue rabbit or a red boat, according to whether they were colored blue or red, or according to their shape. Following a demonstration and two practice trials, the participants completed six trials, initially sorting by color and then by shape. If participants correctly sorted at least five of the six items in both the color and shape sorting phases, they advanced to the third phase: the border task. In this exercise, the children were instructed to sort the cards according to whether they had a black border or a different shape. The participants were informed of the sorting rules before the commencement of each trial. Following the implementation of the revised sorting rule in phases two and three, the number of correctly sorted cards was used to determine the shifting score. This maximum score was set at 12.

***Working Memory*** was evaluated using the EYT “Mr. Ant” Task. The “Mr. Ant” task evaluates participants’ ability to recall the spatial positions of stickers on a cartoon ant. Initially, children view an image of Mr Ant adorned with colored stickers for 5 s, with the number of stickers corresponding to the task's difficulty level. Following a 4-s blank screen, an image of Mr Ant without stickers appears, accompanied by an auditory prompt for participants to recall and indicate the stickers’ locations. The task includes three trials at each level of difficulty, progressively increasing from one to eight stickers. Participants indicated the positions of the stickers by clicking on the corresponding areas on “Mr Ant”. The experiment was concluded either when the subject had completed level 8 (which required the recall of eight positions) or after three unsuccessful trials at the same difficulty level.

***Psychosocial health*** A preschool-specific version of the Social Skills Improvement System Rating Scale (SSIS-RS) was utilized to evaluate children’s social skills and problem behaviors.^36^ This multi-rater scale includes input from teachers, parents/caregivers, and students. In this study, the teacher-reported version was used to assess social skills and problem behaviors. The teacher-reported version of the SSIS-RS demonstrates strong reliability and validity, with item-total correlations exceeding 0.70 and significant associations with established tools such as the BASC-2 (r = 0.78) and Vineland II (r = 0.65).^36^ The Social Skills subscale encompasses the domains of communication, cooperation, assertion, responsibility, empathy, engagement, and self-control. Teachers rated the frequency of each behavior on a four-point Likert scale: “never” (0), “seldom” (1), “often” (2), and “almost always” (3). Higher scores indicate greater social competence. The scale also evaluates problem behaviors, including externalizing behaviors, bullying, and symptoms related to attention deficit hyperactivity disorder (ADHD), autism spectrum disorder (ASD), and internalizing behaviors. Teachers rated the frequency of these problem behaviors using the same 4-point Likert scale (0–3 for never, seldom, often, and almost always, respectively). Higher scores indicate a greater frequency of problem behaviors.

## 5 Statistical analysis

All statistical analyses were conducted using IBM SPSS Statistics (version 26; IBM Corp., Armonk, NY), and SEM was performed using IBM SPSS Amos (version 26; IBM Corp., Armonk, NY). Descriptive statistics, including mean, percentage, and bivariate correlation calculations, were employed to explore the dataset. Mediation analysis was conducted in two stages. First, Pearson correlation coefficients were calculated for all measured variables to identify potential confounding factors. Results showed no statistically significant correlation between BMI and motor skills, executive functioning, or psychosocial well-being; hence, BMI was excluded from further analysis.

Second, a structural equation model (SEM) was estimated using maximum likelihood estimation. Model fit was assessed using several indices: chi-squared (χ^2^) statistic with a degree of freedom (df) ≤ 5; Goodness of Fit Index (GFI) > 0.9; Comparative Fit Index (CFI) > 0.9; root mean squared error of approximation (RMSEA) ≤ 0.08; and standardized root mean square residual (SRMR) ≤ 0.08. These indices followed the guidelines outlined in Ref. 35 Four mediation models were tested: 1) the effect of gross motor skills on social skills via the mediation of executive functions; 2) the effect of gross motor skills on problem behaviors via the mediation of executive functions; 3) the effect of fine motor skills on social skills via the mediation of executive functions; and 4) the effect of fine motor skills on problem behaviors via the mediation of executive functions. Age and sex were included as confounding factors due to influence on motor skills, executive functions, and psychosocial health in young children.^16^^,^^37^^,^^38^ All models were adjusted for age and sex as confounding factors. Statistical significance was set at p < 0.05.

## 6 Results

Table 1 presents a description of the characteristics observed among the study sample. The final analysis included data from 452 children in total, of whom 48.9 % were female. The mean age of participants was 6.14 ± 0.29 years. Table 2 outlines the bivariate correlation coefficients. Both gross (*r* = 0.585, p < 0.01) and fine motor skills (*r* = 0.227, p < 0.01) were positively correlated to social skills. A positive correlation was observed between inhibition and social skills (*r* = 0.429, p < 0.05), while a negative association was identified between inhibition and problem behaviors (*r* = −0.250, p < 0.05). Furthermore, a positive correlation was observed between working memory and social skills (*r* = 0.195, p < 0.05). Additionally, there was a significant negative correlation between social skills and problem behaviors (*r* = −0.568, p < 0.01).

**Table 1:** Descriptive characteristics of the study sample.

Indicator	Mean (n = 452)	SD/%
Sex		
Boys	231	51.1 %
Girls	221	48.9 %
Age	6.14	0.29
BMI	21.45	115.45
Fine Motor Skills	35.98	8.20
Gross Motor Skills	61.46	8.01
Inhibition	0.80	0.14
Shifting	9.66	1.62
Working Memory	3.26	1.11
Social skills	95.18	10.70
Problem behavior	19.92	10.63

Note: BMI: Body Mass Index; SD: standard deviation.

**Table 2:** Bivariate correlation coefficients among gross motor skills, executive functions, and psychosocial health (n = 452).

Indicator	Sex	Age	BMI	FMS	GMS	Inhibition	Shifting	Working memory	Social skills
Age	−0.045								
BMI	−0.047	0.047							
FMS	0.003	−0.113∗	−0.028						
GMS	0.091	−0.198∗∗	−0.039	0.338∗∗					
Inhibition	0.112∗	−0.050	0.017	0.355∗∗	0.414∗∗				
Shifting	−0.047	0.010	0.039	0.164∗∗	0.196∗∗	0.330∗∗			
Working memory	0.022	0.030	0.046	0.212∗∗	0.299∗∗	0.352∗∗	0.286∗∗		
Social skills	0.101∗	−0.164∗∗	−0.076	0.227∗∗	0.585∗∗	0.429∗∗	0.088	0.195∗∗	
Problem behavior	0.075	0.091	0.088	−0.083	−0.280∗∗	−0.250∗∗	−0.001	−0.055	−0.568∗∗

Note: BMI: Body Mass Index; FMS, Fine Motor Skills; GMS: Gross Motor Skills. ∗. Correlation is significant at the 0.05 level. ∗∗. Correlation is significant at the 0.01 level.

### 6.1 Meditation models

**Fig. 1:**
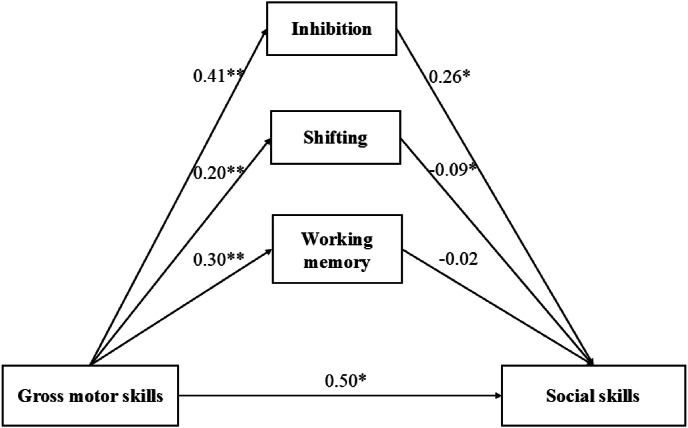
The direct and indirect effects of executive functions in the association between gross motor skills and social skills (∗: *p*-value <0.05; ∗∗. *p*-value <0.01.).

Fig. 1 illustrates the correlation between gross motor skills and social skills. A positive association was observed between gross motor skills (*β* = 0.50, *p* < 0.01) and social skills. Gross motor skills were positively associated with inhibition (*β* = 0.41, *p* < 0.01), shifting (*β* = 0.20, *p* < 0.01), and working memory (*β* = 0.30, *p* < 0.01). Inhibition (*β* = 0.26, *p* < 0.05) was positively associated with social skills while shifting (*β* = −0.09, *p* < 0.05) was negatively associated with social skills. The model fit indices suggest a good fit, with χ^2^(df = 8) = 13.800, χ^2^/df = 1.725, p = 0.087, GFI = 0.991, CFI = 0.988, RMSEA = 0.040, and SRMR = 0.030. Table 3 presents the indirect effects of executive functions on social skills. Inhibition (*β* = 0.107, p < 0.001) and shifting (*β* = −0.018, p < 0.05) mediated the association between gross motor skills and social skills. Memory (*β* = −0.006, p > 0.05) did not mediate this relationship.

**Table 3:** The indirect effects of executive functions in the association between gross motor skills and social skills.

Indirect path	B	Lower	Upper	*p*	*β*
Gross motor skills—Inhibition—Social skills	0.107	0.076	0.151	0.001	0.107
Gross motor skills—Working memory—Social skills	−0.006	−0.027	0.013	0.587	−0.006
Gross motor skills—Shifting—Social skills	−0.018	−0.037	−0.006	0.014	−0.018

Note: B = unstandardized estimates, *β =* standardized estimates.

**Fig. 2:**
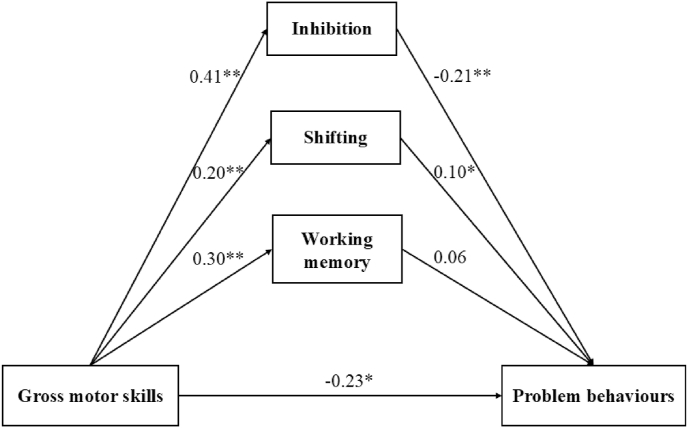
The direct and indirect effects of executive functions in the association between gross motor skills and problem behaviors (∗: *p*-value <0.05; ∗∗. *p*-value <0.01.).

Fig. 2 presents a mediation model that examines the relationship between gross motor skills and problem behaviors. Gross motor skills were positively associated with problem behaviors (*β* = −0.23, *p* < 0.05). Additionally, gross motor skills were positively associated with inhibition (β = 0.41, *p* < 0.01), shifting (*β* = 0.20, *p* < 0.01), and working memory (*β* = 0.30, *p* < 0.01). Inhibition was negatively associated with problem behaviors (*β* = −0.21, *p* < 0.01) while shifting (*β* = 0.10, *p* < 0.05) showed a positive association. The model fit indices suggest an acceptable fit, with χ^2^(df = 8) = 20.105, χ^2^/df = 2.513, p = 0.010, GFI = 0.988, CFI = 0.960, RMSEA = 0.058, and SRMR = 0.034. Table 4 shows the indirect effects of executive functions on problem behaviors. Inhibition (*β* = −0.086, *p* < 0.001) and shifting (*β* = 0.019, *p* = 0.031) mediated the association between gross motor skills and problem behaviors. However, working memory (*β* = 0.018, *p* = 0.196) did not mediate the association between gross motor skills and problem behaviors.

**Table 4:** The indirect effects of executive functions in the association between gross motor skills and problem behaviors.

Indirect path	B	Lower	Upper	*p*	*β*
Gross motor skills—Inhibition—Problem behaviors	−0.086	−0.129	−0.051	0.001	−0.086
Gross motor skills—Working memory—Problem behaviors	0.018	−0.005	0.047	0.196	0.018
Gross motor skills—Shifting—Problem behaviors	0.019	0.004	0.039	0.031	0.019

Note: B = unstandardized estimates, *β =* standardized estimates.

**Fig. 3:**
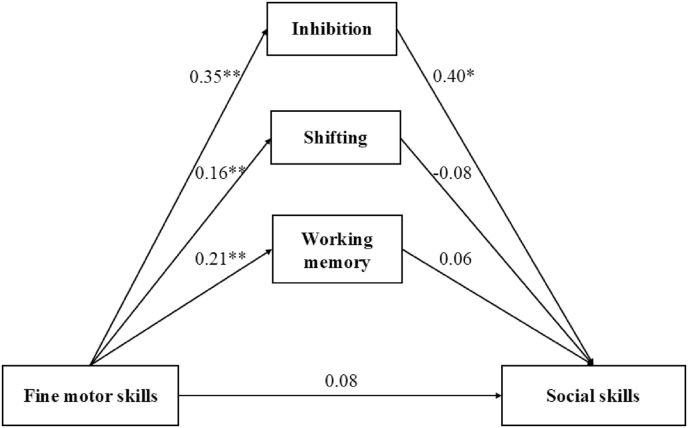
The direct and indirect effects of executive functions in the association between fine motor skills and social skills (∗: *p*-value <0.05; ∗∗. *p*-value <0.01.).

Fig. 3 illustrates a mediation model assessing the relationship between fine motor skills and social skills. Fine motor skills (*β* = 0.08, *p* > 0.05) were not directly associated with social skills. Fine motor skills were positively associated with inhibition (*β* = 0.35, *p* < 0.01), shifting (*β* = 0.16, *p* < 0.01), and working memory (*β* = 0.21, *p* < 0.01). Inhibition was positively associated with social skills (*β* = 0.40, *p* < 0.05), but not for shifting (*β* = −0.08, *p* > 0.05) and working memory (*β* = 0.06, *p* > 0.05). The model fit indices suggest a good fit, with χ^2^(df = 8) = 23.451, χ^2^/df = 2.931, p = 0.003, GFI = 0.986, CFI = 0.949, RMSEA = 0.065, and SRMR = 0.041. Table 5 presents the indirect effects of executive functions on social skills. Inhibition (*β* = 0.144, *p* < 0.001) mediated the relationship between fine motor skills and social skills, whereas shifting (*β* = −0.012, p > 0.05) and working memory (*β* = −0.012, p = 0.161) did not mediate this relationship.

**Table 5:** The indirect effects of executive functions in the association between fine motor skills and social skills.

Indirect path	B	Lower	Upper	*p*	*β*
Fine motor skills—Inhibition—Social skills	0.144	0.106	0.187	0.001	0.144
Fine motor skills—Working memory—Social skills	0.012	−0.002	0.032	0.161	0.012
Fine motor skills—Shifting—Social skills	−0.012	−0.032	−0.001	0.070	−0.012

Note: B = unstandardized estimates, *β =* standardized estimates.

**Fig. 4:**
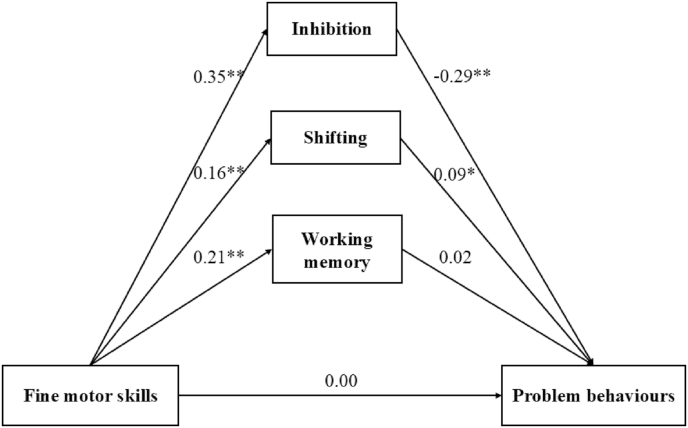
The direct and indirect effects of executive functions in the association between fine motor skills and problem behaviors (∗: *p*-value <0.05; ∗∗. *p*-value <0.01.).

Fig. 4 presents a mediation model examining the relationship between fine motor skills and problem behaviors. The model indicates that fine motor skills (*β* = 0.00, *p* > 0.05) were not directly associated with problem behaviors. However, fine motor skills were positively associated with inhibition (*β* = 0.35, *p* < 0.01), shifting (*β* = 0.16, *p* < 0.01), and working memory (*β* = 0.21, *p* < 0.01). Inhibition (*β* = −0.29, *p* < 0.01) was negatively associated with problem behaviors, while shifting showed a positive association. The model fit indices indicate a good fit: χ^2^ (df = 8) = 21.082, χ^2^/df = 2.635, p = 0.007, GFI = 0.987, CFI = 0.944, RMSEA = 0.060, and SRMR = 0.033. Table 6 presents the indirect effects of executive functions on problem behaviors. The indirect path from fine motor skills to problem behaviors through inhibition (*β* = −0.102, *p* < 0.001) and shifting (*β* = 0.014, *p* = 0.041) mediated the relationship between fine motor skills and problem behaviors. Working memory (*β* = 0.004, *p* = 0.617) did not mediate this association.

**Table 6:** The indirect effects of executive functions in the association between fine motor skills and social skills.

Indirect path	B	Lower	Upper	*p*	*β*
Fine motor skills—Inhibition—Problem behavior	−0.102	−0.144	−0.069	0.001	−0.102
Fine motor skills—Working memory—Problem behavior	0.004	−0.012	0.023	0.617	0.004
Fine motor skills—Shifting—Problem behavior	0.014	0.003	0.033	0.041	0.014

Note: B = unstandardized estimates, *β =* standardized estimates.

## 7 Discussion

This study examined potential relationships between motor skills, executive functions, and psychosocial health in early childhood, with a focus on executive functions as a mediator. Results indicated significant correlations between motor skills and executive functions, and between executive functions (excluding working memory) and psychosocial health. Gross motor skills were significantly correlated with both social skills and problem behaviors. Mediation analyses showed that inhibition and shifting mediated the relationship between gross motor skills and social skills. Inhibition and shifting mediated the relationship between gross motor skills and problem behaviors. Additionally, inhibition mediated the relationship between fine motor skills and social skills, while both inhibition and shifting mediated the relationship between fine motor skills and problem behaviors. We did not find any mediating effects of working memory in preschool children. These findings highlight the role of executive functions in explaining the link between motor skills and psychosocial health in young children, offering valuable insights into this area of research.

Our study found motor skills were positively associated with executive functions in younger children, which aligns with the existing evidence.^5^^,^^39^ A recent meta-analysis suggested that motor competence was moderately correlated to executive functions, with pooled *r* values ranging from 0.18 to 0.34 in young people.^5^ Furthermore, a narrative review of observational studies indicated that the correlation between motor skills and executive functions in preschool children is weak to moderate.^39^ In a meta-analysis focused on typically developing children aged 3–12 years, the strongest correlations were identified between balance, manual dexterity, and executive functions.^15^ For example, a cross-sectional study examining this relationship in Chinese preschool children revealed that overall executive function scores were moderately and positively correlated with fundamental movement skills.^17^ A study by Cook et al. suggested a positive association between gross motor skills and executive functions (i.e., inhibition and working memory) in younger children.^18^ The close relationship between motor skills and executive functions may be attributed to their shared underlying neuroanatomical substrates, including the dorsolateral prefrontal cortex, the cerebellum, and the basal ganglia.^19^^,^^20^ The development of motor skills during early childhood encompasses a range of abilities, including locomotion (such as hopping and jumping), and object control (such as throwing and bouncing).^39^ The acquisition of novel and complex motor tasks also necessitates the involvement of inhibition, shifting, and working memory.^40^ The results of this study provide support for the hypothesis that there is a strong association between motor skills and executive functions in early childhood development.

The results of this study indicate that there is a positive correlation between gross motor skills and social skills in preschool-age children, while there is a negative correlation between gross motor skills and problem behaviors. This result aligns with existing research in this age group.^12^^,^^16^^,^^41^ A recent narrative review on broader health aspects supports the connection between motor skills and social skills, especially in early childhood.^16^ Children who exhibit deficiencies in their motor skills may struggle with social interactions, potentially leading to coping strategies such as reluctance to engage in physical activities.^27^ To illustrate, a prior investigation revealed a robust cross-sectional correlation between motor skills and social skills among young children.^28^ Furthermore, a longitudinal study found that object manipulation skills in the fall were strongly associated with social behavior in the spring.^12^ The existing evidence supports that gross motor skills have benefits for psychosocial development in this population. One potential mechanism is that high motor skills foster a positive cycle of participation, enabling children to engage more in activities that promote socio-emotional skill development.^42^ Nevertheless, our investigation did not reveal a correlation between fine motor skills and psychosocial well-being in preschool-aged children. This finding differs from those of previous studies that posit a relationship between fine and gross motor abilities and psychosocial health in this younger age group.^43^ Nevertheless, it is yet unclear whether fine and gross motor skills have differential impacts on psychosocial health in young children.^44^ One potential explanation for this discrepancy may be attributed to the use of disparate instruments for the evaluation of motor skills in preschool-aged children. For example, the present study utilized the Movement Assessment Battery for Children, Second Edition (MABC-2), whereas earlier research employed the Bruininks-Oseretsky Test of Motor Proficiency, Second Edition (BOT-2)^43^ and the Test of Gross Motor Development, Second Edition (TGMD-2).^44^ Furthermore, it is possible that the association between fine motor skills and psychosocial health is mediated by underlying processes such as executive functions.

The existing evidence suggests a close association between cognitive and psychosocial development. In our current study, we found significant links between inhibition and shifting and psychosocial health (e.g., social skills, problem behaviors) in younger children. Our results align with prior research indicating that executive functions are critical for the development of psychosocial health in preschool children.^45^, ^46^, ^47^, ^48^, ^49^ A meta-analysis has identified a significant association between overall executive functions (effect size = 0.22) and externalizing behaviors in preschool children, and specific associations with inhibition (ES = 0.24), cognitive flexibility (ES = 0.13), and working memory (ES = 0.17).^45^ For instance, a study involving 119 younger children reported significant cross-sectional associations between deficits in executive functions and social competence.^47^ Moreover, evidence from intervention studies indicates that enhancing executive functions can significantly improve psychosocial health in preschool children.^48^^,^^49^

A recent narrative systematic review suggests that motor skills may positively influence social-emotional health in children and adolescents through behavioral, neurobiological, and psychosocial pathways.^16^ However, the role of executive functions as mediators in this relationship has not been formally investigated in younger children. Our mediation analyses suggest that inhibition and shifting mediated the connection between motor skills and psychosocial health. Similarly, executive functions were found to mediate the link between gross motor skills and emotion regulation in early childhood.^30^^,^^31^ Nevertheless, these studies focused on emotional understanding and did not consider social skills or problem behaviors. Furthermore, an intervention study involving both fine and gross motor skills effectively facilitated social skills by enhancing executive functions in preschool children.^48^ However, this intervention did not specifically target motor skills enhancement. In addition, the findings indicated that the link between fine motor skills and psychosocial health is fully mediated by inhibition and shifting in younger children. Despite these findings, the differential effects of fine and gross motor skills on psychosocial health in young children remain unclear.^44^ To gain a comprehensive understanding of these interplays, more studies are needed to explore the role of executive functions as mediators in this relationship in preschool children.

Our findings indicate that shifting does not mediate the relationship between fine motor skills and social skills. One plausible explanation for this finding may lie in the nature of fine motor skill acquisition, which prioritizes precision of hand and finger movements.^50^ Tasks may require precision motor control (e.g., drawing) demand sustained attentional focus and inhibitory control to resist distractions, rather than frequent task-switching. This suggests that inhibition, not shifting, may be the more proximal cognitive mechanism linking fine motor development to collaborative social interactions in younger children. However, given the cross-sectional nature of our study, causal inferences about these pathways remain tentative. Future experimental studies using longitudinal or randomized motor interventions are needed to test the directionality of these associations and disentangle the unique contributions of specific executive functions to psychosoical development.

The absence of working memory as a mediator likely stems from its limited direct association with behavioral outcomes in our sample. This aligns with prior evidence showing that working memory relates reciprocally to internalizing symptoms (e.g., anxiety) but not externalizing behaviors (e.g., aggression) in early childhood.^51^ Three factors may explain this discrepancy. First, teacher-reported behavioral measures may inadequately capture internalizing symptoms, which are less observable than externalizing behaviors. Second, data collection during mid-semester—a period marked by acclimatization to preschool routines—may have suppressed baseline anxiety or depressive symptoms compared to enrollment periods,^51^ thereby attenuating detectable associations. Finally, our analysis did not distinguish between internalizing and externalizing behaviors, potentially obscuring working memory's nuanced role. These limitations collectively underscore the need for multi-method assessments and longitudinal designs to clarify working memory's contributions to behavior regulation.

This study contributes to the existing literature by examining the potential mediating role of executive functions in the relationship between motor skills and psychosocial health in preschool children, an area not yet fully understood. The significant mediating effects highlight the importance of considering the interconnectedness of motor, cognitive, and social development in young children. The “Learning to Learn” framework suggests that motor skill development underpins advanced cognitive abilities and social behaviours.^29^ Social behaviours like self-control, cooperation, and reduced hyperactivity rely on emotional regulation, which engages neural pathways associated with executive function.^10^^,^^11^ Thus, motor skill acquisition can enhance both executive functions and psychosocial development in young children. However, several limitations in this research need to be addressed. First, the use of a cross-sectional design limits the ability to draw causal conclusions. Future experimental studies are needed to clarify these causal relationships. Second, although age and sex were controlled for in the mediation models, future research should consider other potential confounders, such as socioeconomic status (SES) and BMI. As suggested by Hill’s narrative review, the relationships between motor skills, executive functions, and psychosocial health in children are moderated by individual factors (e.g., sex, age, BMI) and environmental factors (e.g., SES).^16^ Third, the study used a product-oriented tool, such as the Movement Assessment Battery for Children (second edition), to assess motor skills, but did not include measures of locomotor abilities. To determine whether the observed relationships between motor, cognitive, and psychosocial development are domain-specific, future studies should incorporate a more comprehensive assessment of motor skills, including both product- and process-oriented measures.

## 8 Conclusion

The results of our study indicate a notable correlation between motor skills, executive functions, and psychosocial well-being in preschool-aged children. Furthermore, it was noted that executive functions mediated the connection between motor and psychosocial development in younger children. The findings suggest that preschool education programs should emphasise not only the development of motor skills but also the enhancement of cognitive control abilities. Strengthening these executive functions may lead to improved psychosocial outcomes and foster overall child development. Further experimental studies are required to establish causal relationships in young children.

## Authorship

All persons who meet authorship criteria are listed as authors, and all authors certify that they have participated sufficiently in the work to take public responsibility for the content, including participation in the concept, design, analysis, writing, or revision of the manuscript. Furthermore, each author certifies that this material or similar material has not been and will not be submitted to or published in any other publication.

The authors whose names are listed immediately below certify that they have NO affiliations with or involvement in any organization or entity with any financial interest (such as honoraria; educational grants; participation in speakers’ bureaus; membership, employment, consultancies, stock ownership, or other equity interest; and expert testimony or patent-licensing arrangements), or non-financial interest (such as personal or professional relationships, affiliations, knowledge or beliefs) in the subject matter or materials discussed in this manuscript.

## Author contribution

Conceptualization, S.L., Z.W.; methodology, S.L., Y. S., Z.W.; investigation, S.L., Z.W.; data curation, S.L., Y. S.; writing-original draft preparation, S.L., Z.W.; writing-review and editing, S.L., Y. S., Q. Z., Z.W.; supervision, Q. Z., Z.W. All authors have read and agreed to the published version of the manuscript

## Data availability statement

Not applicable.

## Funding

This study was supported by Shanghai Education Science Research Project [grant number: C2023112].

## Declaration of competing interest

The authors declare no conflict of interest.
